# Controlling pulsed field ablation efficacy during ablation procedures

**DOI:** 10.1093/europace/euag188

**Published:** 2026-10-21

**Authors:** María Cespón-Fernández, Rafael V Davalos, Thomas Kueffer, Bakhtawar Khan Mahmoodi, Tobias Reichlin, Andrea Sarkozy, Sing-Chien Yap, Edward J Jacobs, Guillaume Gibert, Samuel J Asirvatham

**Affiliations:** Cardiovascular Research Group, Galicia Sur Health Research Institute (IIS Galicia Sur), SERGAS-UVIGO, Department of Cardiology, Hospital Álvaro Cunqueiro, University of Vigo, Vigo, Spain; Wallace H. Coulter Department of Biomedical Engineering, Georgia Tech - Emory University, Atlanta, GA, USA; Department of Cardiology, Inselspital, Bern University Hospital, University of Bern, Bern, Switzerland; Department of Cardiology, Thorax Center, Cardiovascular Institute, Erasmus MC, Rotterdam, the Netherlands; Department of Cardiology, Inselspital, Bern University Hospital, University of Bern, Bern, Switzerland; Heart Rhythm Management Centre, Postgraduate Program in Cardiac Electrophysiology and Pacing, Universitair Ziekenhuis Brussel – Vrije Universiteit Brussel, European Reference Networks Guard-Heart, Brussels, Belgium; Department of Cardiology, Thorax Center, Cardiovascular Institute, Erasmus MC, Rotterdam, the Netherlands; Electrophysiology Department, Boston Scientific Corporation, Marlborough, MA, USA; Electrophysiology Department, Boston Scientific Corporation, Marlborough, MA, USA; Division of Cardiovascular Diseases, Department of Internal Medicine, Mayo Clinic, Rochester, MN, USA

**Keywords:** Pulsed field ablation, Irreversible electroporation, Reversible electroporation, Atrial fibrillation, Lesion durability, Electrogram analysis, Catheter ablation, Intracardiac echocardiography, Electroanatomical mapping, Ablation indexes, Contact assessment

## Abstract

Pulsed field ablation (PFA) has rapidly emerged as a promising ablation modality for the treatment of cardiac arrhythmias because of its myocardial tissue preferential ablation and favourable safety profile. However, procedural control of lesion formation and reliable assessment of lesion durability remain important challenges. This review summarizes the main determinants of PFA efficacy and current approaches for intraprocedural lesion assessment. Experimental and clinical studies indicate that PFA lesion formation results from the interaction between delivered electrical energy, catheter design, tissue coupling, and application strategy. Lesion size and geometry are primarily determined by electric field distribution and cumulative electroporation exposure, while excessive energy delivery may induce unwanted thermal or electrochemical effects. Procedural efficacy also depends on stable catheter positioning, adequate tissue proximity, and sufficient lesion overlap. Unlike thermal ablation technologies, conventional procedural markers such as electrogram attenuation, impedance changes, and pacing threshold variation correlate poorly with durable irreversible electroporation. Multiparametric lesion estimation models integrating electrical, mechanical, and positional data have shown encouraging preclinical results, although clinical validation remains limited. Emerging strategies combining electroanatomical mapping, imaging, and automated electrogram analysis may improve real-time evaluation of lesion continuity and durability. Current PFA workflows rely mainly on standardized energy delivery protocols, optimized catheter positioning, and lesion overlap rather than real-time titration of energy. Although robust intraprocedural markers of irreversible electroporation are still lacking, ongoing advances in catheter technology, imaging, and lesion assessment tools are expected to improve procedural reproducibility and long-term lesion durability. Further studies are needed to establish validated endpoints for durable PFA lesion formation.

## Graphical Abstract

**Graphical Abstract:**
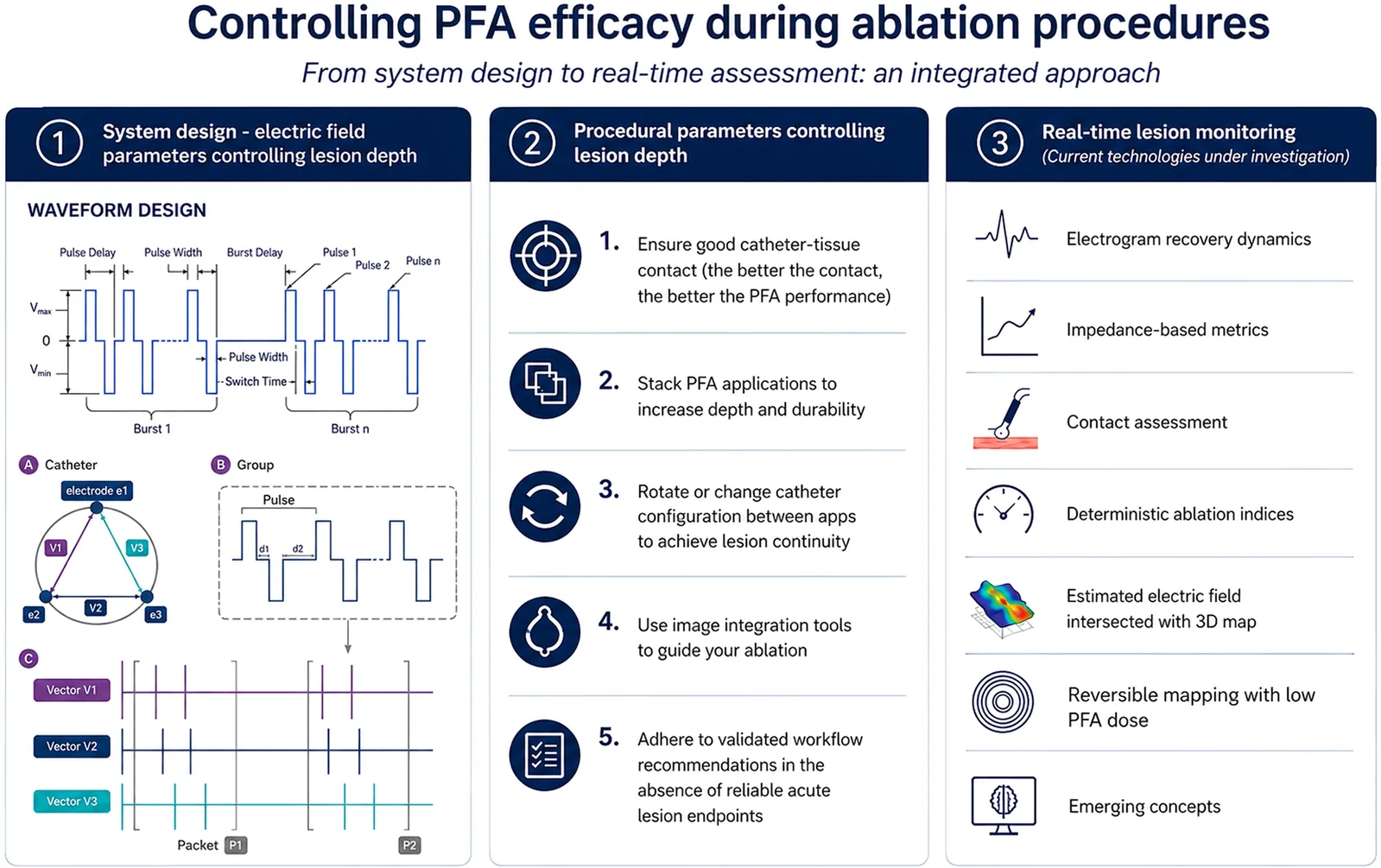

## Introduction

The goal of pulsed field ablation (PFA) is to preferentially eliminate or electrically isolate aberrant cardiomyocytes through irreversible electroporation while avoiding thermal injury to adjacent critical structures. Therefore, the primary targets for treatment design are safety and efficacy. Secondary targets include efficiency, as faster and easier treatments can improve the accessibility and throughput of clinicians. Since the clinical outcomes (i.e. lesion area) and by-products (i.e. Joule heating, haemolysis, bubbles, pain, and muscle stimulation) are both functions of the catheter and recipe,^1^ no ubiquitous protocol has been established to date. The catheter shape, tissue type and health, contact, and waveform all influence the electric field distribution within the tissue, but the thresholds for inducing electroporation and lethal cell death are only dependent on the waveform and the tissue.^2^ Different tissues can have distinct irreversible electroporation thresholds that depend on cellular size and orientation, local conductivity, and sensitivity to homeostatic disruption. Therefore, catheter and recipe design are symbiotic, with each unique catheter potentially possessing different ideal waveforms and delivery vectors for its use cases. Unlike thermal ablation, where tissue injury develops immediately around the electrode and several procedural surrogates (e.g. impedance drop, electrogram attenuation, temperature, and contact force) can help estimate lesion formation, PFA produces a more complex and dynamic biological response. Exposure to pulsed electric fields generates a central zone of irreversible electroporation surrounded by a peripheral region of reversible electroporation, in which cardiomyocytes may transiently lose excitability and conduction but ultimately recover membrane integrity and function. Consequently, acute conduction block or electrogram attenuation (or disappearance) does not necessarily indicate permanent tissue injury, as transient electrical silence may simply reflect reversible membrane permeabilization rather than irreversible cell death. The extent of this reversible penumbra depends on the local electric field distribution, waveform characteristics, catheter configuration, and tissue properties, while its recovery kinetics are influenced by the severity of electroporation. Furthermore, unlike thermal coagulation necrosis, irreversible electroporation initiates a cascade of biological processes (including disruption of membrane homeostasis, ionic imbalance, metabolic failure, and regulated cell death pathways) that continue to evolve after energy delivery. As a result, acute procedural endpoints may not accurately reflect chronic lesion durability, and clinicians currently lack a validated intraprocedural marker capable of distinguishing reversible from irreversible electroporation or reliably predicting long-term lesion permanence. This limitation shifts procedural optimization towards understanding the biophysical determinants of lesion formation and identifying procedural parameters that maximize the likelihood of durable irreversible injury.

This complexity raises a central procedural question: in the absence of direct methods for measuring lesion formation, what parameters can the operator meaningfully control during a PFA procedure to influence efficacy, and how can procedural decisions be guided to ensure that the intended biological effect is achieved? This article explores the mechanisms, limitations, and practical levers available to clinicians for controlling PFA efficacy in real time.

## Electric field parameters controlling ablation efficacy

### Factors increasing lesion depth

While specific parameter values for commercial PFA systems are not disclosed, the waveforms are typically comprised of the same components, with different nomenclatures.^2–4^ One application of PFA treatment consists of a train of packets, each containing a series of biphasic square pulses (*Figure 1*). The biphasic square pulses are separated by interphase and interpulse delays and repeated for several cycles. For multielectrode probes, only one electrode pair is typically active at a time; therefore, this pulse series is repeated for a defined sequence of electrode pairings (the vector) to form the packet. From early PFA implementation, packet delivery was typically synchronized with the R-wave of the paced heart rate (∼500 ms).^5,6^ Although asynchronous delivery is now commonly utilized, the rate of packet delivery has been conserved.^7^ The train of delivered packets constitutes the application, with different anatomical locations sometimes receiving different application counts, typically due to differences in tissue thickness. For example, the pentaspline catheter delivers eight applications per pulmonary vein, whereas only two applications are delivered at each location along the left atrial (LA) posterior wall.^8^

**Figure 1:**
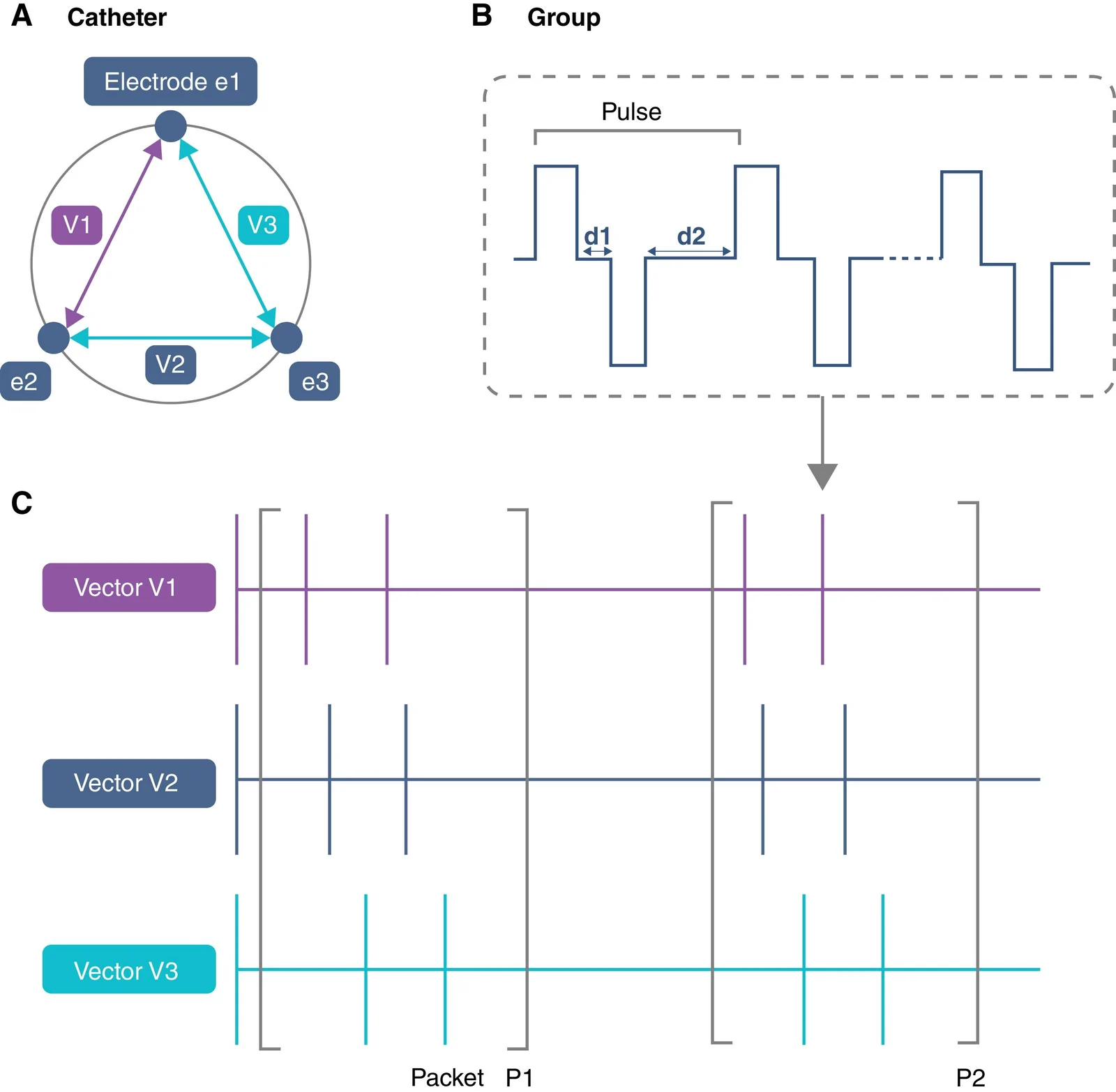
*A*) Defined vectors delivered between catheter electrodes. *B*) A singular group with biphasic pulses separated by interphase (d1) and interpulse (d2) delays. *C*) A single packet, P, is typically delivered per heartbeat and is assembled by vectors that deliver groups in specified orders. The series of predefined packets, P, constitutes an application.

Among waveform variables, lesion depth is primarily governed by peak electric field magnitude and cumulative time above the critical transmembrane potential, with pulse width and pulse number acting as coupled modulators. Timing parameters (interphase, interpulse, and interpacket delays) exert secondary effects unless they alter total application duration. Pulse width has a positive logarithmic correlation with lesion size,^9–12^ and values ranging from 350 ns to 100 µs have been shown to generate cardiac lesions in preclinical models.^13,14^ The use of short pulses around or below the cell membrane charging time (0.5–2 µs) reduces the influence of cell membrane capacitance on current flow, providing more uniform and predictable lesions and mitigating neuromuscular excitation.^15–17^ However, with shorter pulse widths, the portion of the membrane that exceeds the critical transmembrane potential is exposed for a shorter time.^18,19^ Pore formation and subsequent mass transport then decrease, leading to smaller areas of irreversible electroporation.^9–11^ Thus, longer pulse widths remain more electroporatively efficient per pulse, whereas shorter pulses trade efficiency for predictability and safety. To increase lesion sizes at short pulse widths, higher local electric fields must be generated by increasing the waveform magnitude to increase the rate of electroporation within the shorter time frame.^20^ The number of pulses can also be increased to extend the total duration during which the membrane is above the critical transmembrane potential. However, increasing the waveform magnitude and the number of pulses both result in additional Joule heating and electrolytic effects.^21–23^ Joule heating is not affected by parameters equally, scaling quadratically with waveform magnitude and linearly with duration and pulse number.

As a result, increasing pulse number is generally a more thermally efficient strategy for lesion enlargement than increasing amplitude, provided neuromuscular and electrolytic limits are respected. Increasing the separation between packets may also allow for thermal diffusion to reduce maximal temperatures during treatments at the expense of application time. Applying the same number of pulses over a longer period has also been shown to extend the pore lifetime, improving total transmembrane mass transport and moderately increasing lesion size.^24,25^ The distinction between packet separation and pulse separation has not been fully elucidated, but it appears that the lesion will be affected if either separation extends the overall application time.^26^ Clinically, this distinction is relevant because packet timing and numbers are often constrained by requirements for rapid delivery times, whereas interpulse timing is a waveform design variable. Experimental data demonstrate that cardiac lesions are not affected by interphase and interpulse delays when the application length is maintained.^9^ Computational models of neuromuscular excitation suggest that shorter interphase delays and longer interpulse delays theoretically decrease neuromuscular excitation,^27^ but experimental results showed mixed results, with shorter interphase delays only marginally decreasing neuromuscular excitation and pain during PFA delivery.^28,29^

### Cumulative dose and saturation effects

While increasing the number of pulses through waveform adjustments or additional applications generally increases lesion size, electroporation effects saturate.^11,30,31^ In PFA, dose is not a single controllable variable but instead reflects the cumulative exposure of tissue to supra-electroporative field, where higher electric fields increase the rate of mass transport and, subsequently, decrease the time for disruption of homeostasis. The formation of pores creates alternative ion pathways, significantly increasing cardiac tissue conductivity.^10,16^ When subsequent pulsed electric fields are applied, the current preferentially enters the now lower-impedance tissue, thereby extending the reach of the cumulative electrical field and region of mass transport.^32^ However, as electroporation progresses, conductivity-mediated field redistribution equilibrates, and additional pulses no longer expose new tissue volumes to supra-electroporative fields. The timescale and pulse number for this saturation are not yet clear in cardiac tissue, but at this point, further increases in pulse number primarily reinforce already electroporated regions rather than expanding the zone. Although this conceptual framework is supported by experimental and computational studies, the mechanisms governing saturation of lesion expansion remain incompletely characterized in human and diseased myocardium, requiring further clinical validation.

In parallel, cellular permeability is not uniform across the entire electroporated region, as cells at the periphery experience reduced transmembrane mass transport compared with those at higher electric fields closer to the electrodes. Also, regions initially reversibly electroporated can, upon repeated PFA pulses, experience pore enlargement, increased pore number, or prolonged pore stability. Consequently, areas that would have recovered after a single application may undergo irreversible electroporation after repeated exposure, even at the same applied field intensity and independently of local conductivity changes induced by the first application. In this way, cumulative membrane destabilization converts previously reversible regions into permanent lesions.

## Catheter design parameters controlling lesion characteristics

### Procedural parameters controlling ablation efficacy

#### Catheter position, proximity, contact, and force

##### Effect of proximity and contact

Contact force has historically been used to verify apposition and to predict lesion depth with thermal modalities,^33,34^ but it has played a more limited role in PFA. In radiofrequency ablation (RFA), increasing force directly augments resistive heating and conductive heat transfer, yielding a strong correlation with lesion size and depth. In contrast, PFA lesion formation depends on whether the local electric field exceeds a critical electroporation threshold, making electrical parameters the dominant drivers of tissue effects. However, adequate contact is still required to ensure stable electrode–tissue apposition, and insufficient force can cause catheter movement that shifts the effective field within the tissue, increasing the likelihood that portions of the target tissue are exposed to electric fields below the threshold for irreversible electroporation, resulting in incomplete lesion formation. Small increases in electrode–tissue spacing on the order of millimetres will also reduce the local field strength, as the entire field distribution shifts away from the tissue and into the blood pool.^35,36^ Consequently, shunting current into the blood rather than into tissue increases the potential for bubble formation and haemolysis, so a minimum contact-force threshold is necessary to consistently generate the field within the targeted areas^30,37,38^ for both reliable lesion efficacy and safety.

Impedance-based contact and proximity indicators have emerged as a practical surrogate for assessing electrode–tissue apposition:^39–41^ because cardiac tissue is more resistive than blood, the PFA catheter can deliver low-voltage bipolar electrical signals between its electrodes to determine the impedance at the treatment electrodes. The sensitivity of measurements is proportional to the current density within the sample relative to the current leaving the catheter, so measurements are often superficial to the electrode surface where the current density is highest. Further, contact impedance is often limited to bipolar configurations, as the noise from current passing through extra-cardiac regions would outweigh the contact signal at the electrode surface in monopolar configurations. However, the contact measurement electrode pairs do not necessarily need to match the treatment pairs, thereby allowing monopolar ablation with bipolar sensing. Although absolute impedance values are influenced by factors such as anatomical location, tissue disease state (i.e. fibrosis and oedema), and haematocrit, limiting their standalone utility, sampling impedance relative to blood pool measurements provides a more reliable indicator of proximity.^39,42,43^ These impedance-based metrics can be integrated into indices of tissue proximity to guide ablation delivery, improving lesion continuity while avoiding excessive engagement or energy deposition into blood. Yamashita *et al.* indicated an impedance change of >10% predicted adequate pentaspline (Farapulse) catheter contact, which subsequently reduced pulmonary vein gaps in a contact index-guided prospective cohort.^39^ The authors also proposed that very large impedance increases may indicate deep catheter seating within structures such as the pulmonary veins, where slight withdrawal can optimize positioning and reduce unintended tissue injury.

##### Is force predictive of lesion depth?

While the importance of catheter apposition has been repeatedly demonstrated, the influence of additional contact force on lesion depth is less clear. Most studies report significant increases in lesion depth with increasing contact force,^31,44–47^ while others show a minimal but positive influence.^37,48,49^ The versatile nature of PFA has enabled a wide range of catheter designs, from small focal tips to large baskets and paddles. Certain design parameters, particularly electrode size and positioning relative to the tissue surface, may influence how a catheter responds to contact force. Mattison *et al*. reported an increase of 0.01 mm in lesion depth per gram of applied force using a 4.1 mm diameter focal catheter.^28^ One mechanism by which force can influence lesion depth differently across catheter designs is mechanical tissue compression. Higher force may allow deeper tissue layers to experience lethal electric fields, not by increasing field strength but by shifting tissue closer to a fixed electric field distribution. For small tip focal catheters, force is not required to establish contact between the tip electrode and tissue surface (i.e. contact can be reached at 0 g force), while for multielectrode catheters like baskets, ensuring uniform contact with all electrodes placed on multiple sides may require an additional force to deform the tissue surface and reduce the electrode-to-target distance.

While force has primarily been studied with small focal-tip catheters, its role in larger-footprint catheters is less straightforward. In these systems, the applied force is distributed over a larger surface area, meaning that local pressure, rather than total force, governs tissue deformation and electrode contact. Consequently, measured force may be a poor surrogate for effective contact unless sufficiently high levels are applied to ensure consistent electrode–tissue engagement across the entire array. With force-sensing focal-tip catheters, the force sensor and the ablating electrode are co-located, meaning that the measured force closely reflects the actual contact between the electrode and the tissue. In this context, increases in contact force provide a relatively accurate indication of electrode–tissue interaction and positioning.

At the same time, excessive force may introduce treatment variability by distorting tissue or catheter geometry into non-physiological or unvalidated configurations. These proposed mechanisms are primarily derived from preclinical observations and engineering principles; their relative contribution across different clinical catheter platforms remains to be established. Overall, the role of contact force in PFA is best understood as a tool to guide catheter positioning, maintain apposition, and, in some cases, enhance tissue engagement through compression. Although its impact is evident in focal catheter studies, it appears less pronounced than in RFA. Therefore, the integration of contact force into catheter design and lesion prediction models should be balanced against other influential parameters, such as voltage and number of applications. Additionally, excessive force (particularly with small or sharp electrodes) may increase the risk of perforation, highlighting the need to define catheter-specific safe and effective force ranges.

#### The role of imaging integration

For atrial fibrillation ablation, the use of intraprocedural imaging depends on multiple factors, including the specific PFA system employed, the availability of imaging modalities, the experience of the operator and the team, and associated costs. Over-the-wire PFA catheters [e.g. pentaspline catheter (Farawave), circular multielectrode catheter (PulseSelect), and balloon-in-basket catheter (Volt)] are typically used in conjunction with a fluoroscopy-based workflow, while other PFA catheters [e.g. variable loop circular catheter (Varipulse) and lattice-tip catheter (Sphere9)] necessitate integrated 3-dimensional electroanatomic mapping (3D-EAM) systems for catheter positioning and navigation. Among these, the pentaspline ablation catheter is the most widely used, and most evidence regarding the incremental value of intraprocedural advanced imaging pertains to this catheter.

##### Fluoroscopy

Fluoroscopy, with or without pulmonary vein (PV) angiography, has remained the primary modality for guiding single-shot PFA in many centres, particularly when costs, infrastructure, or training pose limitations on the use of advanced imaging technologies (*Figure 2*).^8,50^ Main reasons for not using 3D-EAM systems are additional costs, procedural time, and perceived lack of added value.^50^ Coaxiality and contact of the PFA catheter to the PVs are important for achieving optimal ablation lesions. For the pentaspline catheter, a practical guide was recently published providing tips for a primarily fluoroscopy-based workflow.^8^ Using fluoroscopy, electrode–tissue contact can be assessed by the slight squeeze of the splines in the basket pose or by the slight backflip sign in the flower pose. Fluoroscopy can also be used to guide the pentaspline catheter to targets beyond PVs, including the LA roof, posterior wall, cavotricuspid isthmus, and superior vena cava.^8,51^ However, there are concerns regarding the durability of ablation lesions when the pentaspline catheter is used for mitral isthmus ablation, coronary sinus, and LA appendage isolation.^52^ Despite the widespread use of fluoroscopy, there are significant inherent limitations to fluoroscopy-based ablation. Specifically, fluoroscopy offers no tissue detail, does not provide direct visualization of myocardial contact, and exposes both the patient and operator to radiation hazards.

**Figure 2:**
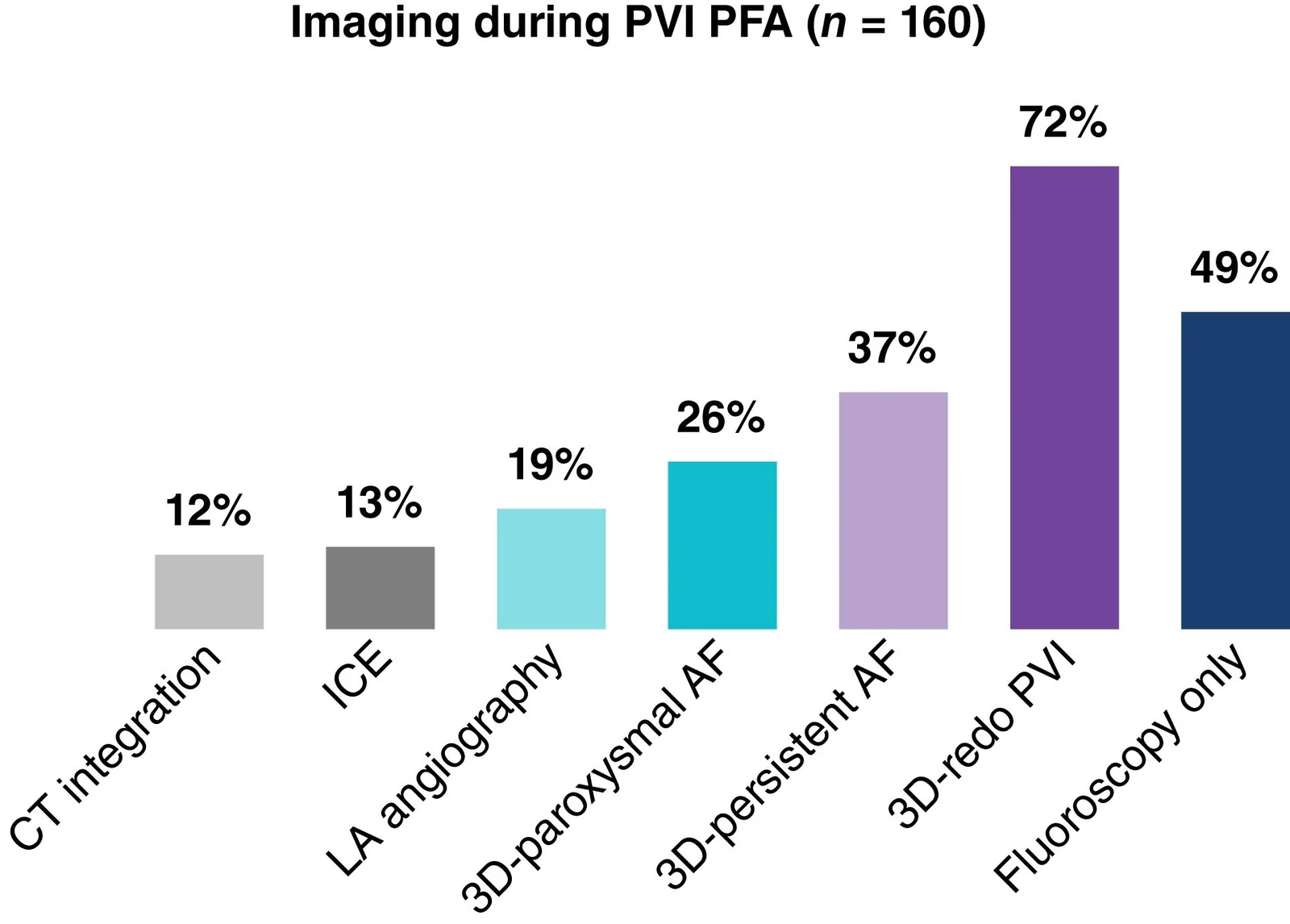
2025 EHRA survey detailing intraprocedural workflows with respect to imaging. During the procedure, half of the 160 respondents relied solely on fluoroscopy, while the remainder variably used 3D electroanatomical mapping systems, intracardiac echocardiography, and atrial angiography. Adapted from Bergonti *et al*.^1^ 3D, three-dimensional; CT, computed tomography; ICE, intracardiac echocardiography; LA, left atrium; PFA, pulsed field ablation; PVI, pulmonary vein isolation.

##### Intracardiac echocardiography

Intracardiac echocardiography (ICE) addresses one of the major limitations of fluoroscopy-based ablation: the inability to reliably visualize catheter-tissue contact. By providing high-resolution, real-time imaging of intracardiac anatomy, catheters, and sheaths, ICE ensures better catheter-tissue contact (*Figure 3*), as demonstrated for the pentaspline catheter, ultimately reducing arrhythmia recurrence and reconnection rates.^53,54^ Intracardiac echocardiography offers other benefits, such as guiding transseptal access, handling anatomical variations of the pulmonary veins, monitoring catheter stability during pulse delivery, and detecting complications like pericardial effusion or thrombus formation.^8^ However, despite these advantages, ICE does not seem to improve other procedural metrics such as procedure time, fluoroscopy time, and number of PFA applications.^53,54^ Due to costs and reimbursement limitations, ICE remains less widely used in Europe compared with the USA.^50^

**Figure 3:**
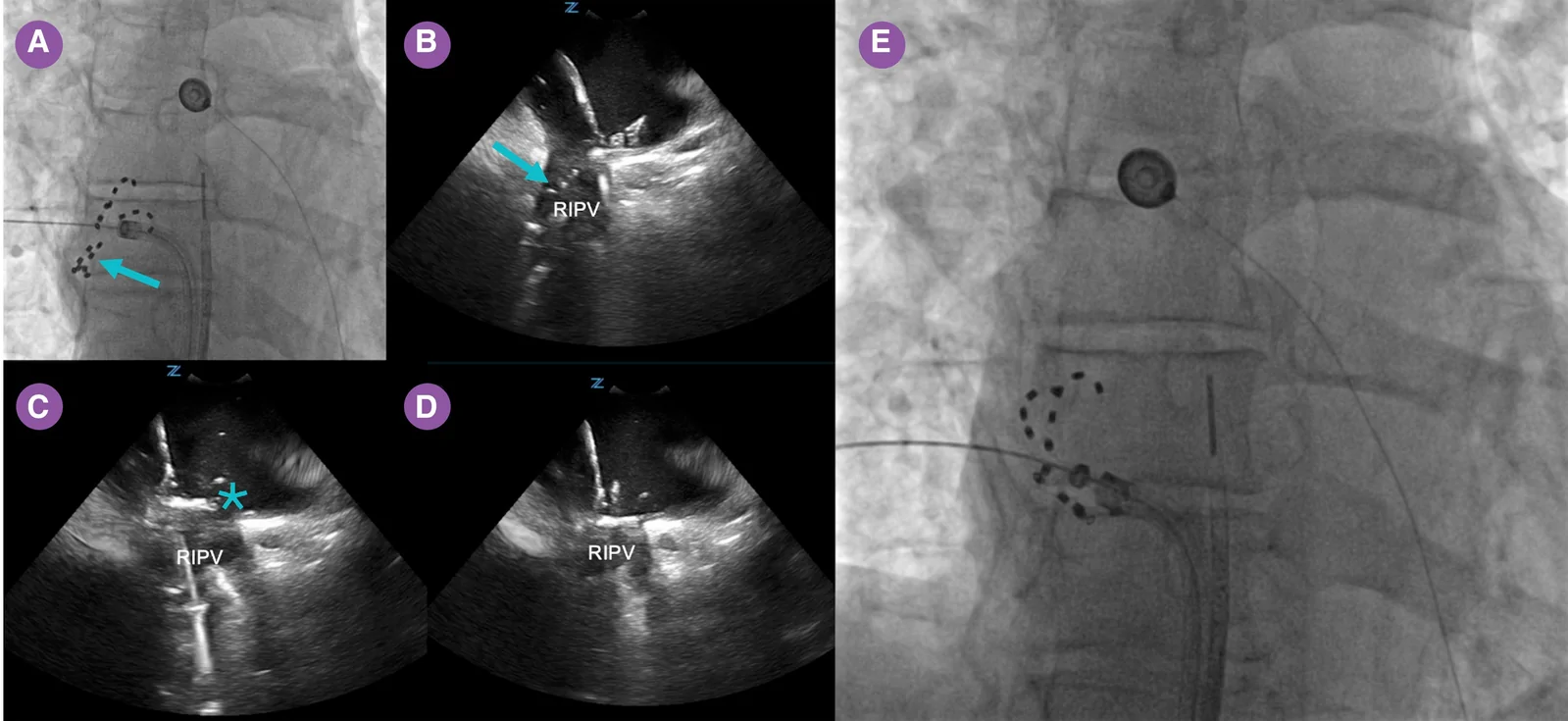
Assessment of catheter-tissue contact using ICE. *A* and *B*) Two petals of the pentaspline catheter (arrow) are in the RIPV as demonstrated by fluoroscopy and ICE imaging. *C*) After gentle clockwise rotation, the petals are rotated outside the RIPV, but there is suboptimal contact (asterisk). *D* and *E*) After advancement of the pentaspline catheter and the sheath electrode–tissue contact is optimized as demonstrated by ICE imaging (*D*) and the backflip sign of the petals on fluoroscopy (*E*). Images are courtesy of Dr Khan Mahmoodi (Erasmus MC). ICE, intracardiac echocardiography; RIPV, right inferior pulmonary vein

##### 3D Mapping

By providing detailed 3D anatomical visualization, precise catheter location tracking, and the ability to perform 3D activation and voltage mapping, 3D-EAM systems help minimize the reliance on fluoroscopy and ICE during ablation. Impedance-based, open-platform 3D-EAM systems (e.g. EnSite X or EnSite Precision) allow for real-time visualization of the pentaspline or circular multielectrode catheter, aiding in the precise navigation and guidance of atrial fibrillation ablation.^55–57^ To maximize the accuracy of these systems, optimizing visualization parameters is crucial to ensure the most accurate anatomical geometry.^58^

Unlike thermal-based lesion assessments, where operators can rely on changes in electrograms, impedance, and temperature, PFA lesion assessment is more challenging. For successful creation of contiguous overlapping lesions, it is essential to precisely track pulse delivery. 3D-EAM systems provide this vital information, allowing for the accurate visualization of PFA lesion location and extent. Several fully integrated mapping platforms (e.g. navigational pentaspline catheter in the OPAL HDx mapping system, lattice-tip catheter in Affera Prism-1, variable loop circular catheter in CARTO3, and balloon-in-basket catheter in ENSITE) enable precise tracking of lesion areas. For example, when the navigational pentaspline catheter is used in conjunction with the FARAVIEW software of the OPAL HDx system, the system can automatically display the electric field relative to the anatomical shell, with automated tags that have demonstrated good correlation with chronic post-ablation low-voltage areas.^7^

Recently, the acquisition of the 3D anatomical shell can also be done by ICE imaging. For example, artificial intelligence-assisted ICE mapping, such as the CARTOSOUND FAM Module, can reconstruct detailed 3D LA anatomy from two-dimensional ICE clips, typically obtained from the right atrium and right ventricular outflow tract (*Figure 4*), enabling even zero-fluoroscopy procedures.^59^ When combined with the variable loop circular catheter platform, this approach has been shown to significantly reduce mapping time, LA dwelling time, and fluoroscopy time compared with conventional 3D mapping.^60^ Despite these advancements, formal evaluation of the long-term efficacy and cost-effectiveness of integrating ICE with 3D-EAM systems is still needed.

**Figure 4:**
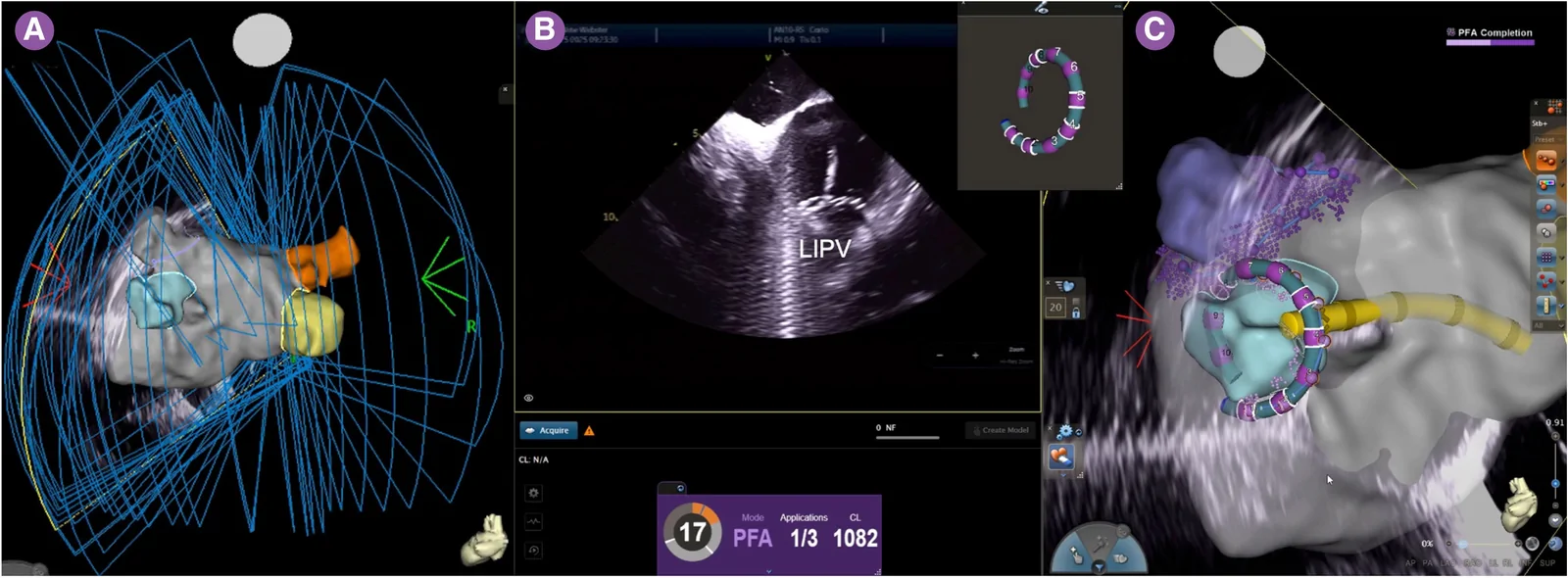
3D anatomical mapping using the CARTOSOUND FAM MAP Module. *A*) Using the SOUNDSTAR ICE catheter, several frames are acquired from each left atrial structure, including the left atrial appendage and pulmonary veins, both from the right atrium and right ventricular outflow tract. Using a deep learning algorithm, an anatomical FAM shell is reconstructed from the acquired frames. *B* and *C*) This reconstructed shell can be used to navigate the variable loop catheter towards the antral side of each pulmonary vein. There is a good correlation of the position of the PFA catheter on ICE imaging (*B*) and the FAM shell (*C*). Images are courtesy of Dr Yuri Blaauw (UMCG). ICE, intracardiac echocardiography; PFA, pulsed field ablation

### Dosing and workflow optimization considerations

Pulsed field ablation produces myocardial injury through electroporation, creating a central irreversible electroporation zone surrounded by a reversible electroporation border. Reversible electroporation can transiently suppress excitability and conduction yet recover, whereas irreversible electroporation leads to delayed cell death and durable lesion formation. From a dosing perspective, this means that acute electrogram suppression is necessary but not sufficient. The practical goal is to reliably convert enough of the intended target volume from reversible into irreversible electroporation while maintaining spatial continuity around the PV antra.

#### Stacking applications: why additional pulses can deepen lesions

A key dosing lever in PFA is stacking lesions by repeating pulse packages and stacking applications in the same catheter position. Preclinical data support that lesion dimensions depend on application number. In a ventricular model, lesion formation correlated with application number and contact force and was approximately linear for low numbers of applications but plateaued with higher numbers/contact force.^44^ A separate investigation using a monopolar ablation vector showed a similar relationship.^30^ In yet another, authors show a clear dose-response relationship for both monopolar and bipolar ablation vectors and that monopolar waveforms may reach deeper into the tissue.^61^

#### From biophysics to workflow: overlap for homogeneity, stacking for depth

Workflow optimization must solve two problems simultaneously: homogeneity (avoid gaps) and adequate depth. Morphometry work with the pentaspline catheter provides a practical rationale for rotation: depending on catheter pose, single applications can produce linear lesions with gaps, while repeating applications and using different orientations increase circumferential continuity and lesion width.^62^ This translates clinically into delivering applications with rotations between applications and ensuring overlap across splines. In addition, extra applications in the carina region ensure confluent periosteal lesions.

Clinical protocol extensions follow the same logic. The ‘olive strategy’ adds two applications in a smaller olive-shaped configuration, increasing dosing from 8 to 10 applications per PV. In 400 consecutive patients treated with this strategy, redo remapping showed PV reconnection in 13.6% (3 of 22 patients) compared with 45% (36 of 80 patients) after a conventional strategy (*P* = 0.007)^63^ At the other end of the spectrum, simplified protocols with fewer total applications have been reported in select cohorts, but small redo cohorts limit firm conclusions.^64^ As additional PFA applications typically prolong procedures by only a few minutes, workflow optimization should focus on lesion continuity and durability rather than marginal time savings, with the understanding that optimal protocols are platform-specific and evolve with experience.^65,66^

As additional single-shot platforms and emerging true single-shot concepts mature, platform-specific dosing and optimized workflows will remain central because in PFA, durable outcomes are shaped as much by where and how energy is delivered (coverage, overlap, and tissue coupling) as by how many applications are applied.

### Limitations on titration capability

In PFA, effective dosing is largely achieved through repetition of applications rather than continuous adjustment of waveform parameters. This reflects both the threshold-based nature of electroporation and system-level safety constraints. Incremental changes in application number or parameter modifications therefore do not translate into proportional or predictable changes in lesion depth or width.

From a biophysical perspective, deeper electroporation can be achieved by increasing electric field strength through higher voltages or longer effective pulse trains. However, both approaches increase thermal loading at the electrode–tissue interface, promoting microbubble formation, electrochemical reactions, and arcing. Minimizing gas formation favours biphasic trains of short microsecond pulses, substantial interpulse delays (low duty cycle), and electrode designs that avoid high local current densities.^1,23^ As a result, maximum usable voltage amplitude, pulse width, and duty cycle are constrained by safety considerations. Achieving deeper lesions therefore depends more on catheter and waveform designs that can safely sustain higher field strengths than on indefinitely stacking applications at the same site.

These constraints shift titration from the operator to the system designer. Voltage amplitude, pulse width, pulse number, and interpulse delay must be optimized for a specific catheter geometry and electrode configuration to control current density and heating. Once validated, these parameters are largely fixed, leaving the operator with limited adjustable variables. This contrasts with RFA, where power, temperature targets, duration, and irrigation can be modified and interpreted using established lesion indices.^1^

Consequently, practical titration in PFA is reduced to a discrete set of procedural controls: application count, catheter pose and orientation, overlap and rotation strategy, and electrode-to-tissue coupling. For this reason, titration by increasing delivered ‘energy’ is not a meaningful clinical concept in PFA. Durable lesion formation instead relies on protocolized application repetition and optimized catheter positioning and coverage.

#### Lack of real-time lesion feedback

A further limitation is the absence of a widely accepted real-time lesion quality marker for PFA comparable to radiofrequency surrogates such as impedance drop, electrogram attenuation, or interval prolongation. Acute effects may reflect reversible electroporation and transient conduction suppression, meaning that acute block does not necessarily indicate durable lesion formation.

Preclinical data underscore this limitation. In a swine study targeting specific lesion depths using a PFA index derived from contact force and pulse count, intracardiac electrogram attenuation, impedance decrease, and electrode temperature showed little or no correlation with lesion size, whereas an index incorporating contact force and pulse count predicted lesion depth with high accuracy.^67^ Ultimately, effective PFA lesion formation likely depends on catheter-to-tissue coupling, where higher contact force, in the case of a focal PFA catheter, might help improve stability and reduce intermittent contact loss during cardiac motion in the beating heart, even if the influence of force in stationary models remains debated. Notably, contact force remains the most reliable surrogate of lesion formation in RFA, outperforming electrogram attenuation, impedance changes, and other commonly used real-time markers.^68,69^

#### Practical implication

In summary, PFA titration is limited because lesions are delivered as short waveforms, platform parameters are fixed by design to balance efficacy with safety constraints such as heating and gas formation, and real-time feedback markers that reliably track lesion size are still evolving. As a result, contemporary PFA workflows favour standardized protocols and discrete procedural adjustments rather than continuous titration of energy delivery.

## Real- time monitoring of ablation success in pulsed field ablation

### Multiparametric estimators

#### Current knowledge and limitations

Ablation indexes have been developed to standardize lesion delivery and improve procedural reproducibility in catheter ablation workflows. In RFA, composite metrics such as the ablation index (AI) have demonstrated utility in estimating lesion depth and have been incorporated into standardized protocols (e.g. CLOSE protocol^70^) to guide lesion delivery and interlesion distance. These approaches have contributed to workflow harmonization across centres and operators.

In the context of PFA, several indexes have been proposed^30,31,44^ to estimate lesion characteristics based on procedural input parameters, including contact force, applied voltage, and the number of pulse trains or applications. Preclinical studies have reported correlations between such indexes and lesion depth or geometry.^71^ However, these indexes remain inherently limited by factors that influence the actual electric field distribution and tissue response, which are not fully captured by procedural input parameters alone.

Multiple determinants affect lesion formation in PFA, including catheter orientation, tissue–electrode coupling, tissue composition, and myocardial anisotropy.^30,72^ Tissue properties such as fibrosis, ischaemia, and adipose infiltration alter electrical conductivity and susceptibility to electroporation, thereby influencing lesion size and transmurality. Consequently, the current delivered to the myocardium may differ substantially from that predicted by nominal system settings.

#### Future perspectives

The concepts discussed in this section represent emerging approaches intended to improve procedural guidance. Unless otherwise stated, these strategies remain investigational and have not yet been validated for routine clinical decision-making. Recent work has explored composite PFA indexes incorporating voltage amplitude, contact force, catheter orientation, and the number of applications to estimate lesion geometry (depth and width).^30^ This study has reported correlations between such composite index and lesion depth in experimental models.

Additionally, interdependencies among procedural parameters have been identified. For example, the incremental effect of repeated applications on lesion depth appears to depend on the applied voltage, with limited additional depth observed at lower voltage levels. Low-voltage PFA produces smaller reversible and irreversible zones due to the lower electric field gradient, whereas high-voltage PFA generates larger zones of both types. When a second application is delivered, part of the reversible zone can be converted into irreversible injury. Because high voltage creates larger reversible zones, this conversion results in a proportionally greater increase in irreversible damage. Similarly, the relative importance of contact force may decrease with increasing number of applications, suggesting that limited contact force can be partially compensated by repeated pulse delivery. Conversely, contact force may play a more significant role at lower voltage levels.

These interdependencies indicate that single-parameter or purely additive models may not accurately predict lesion depth and that multivariable interactions should be considered when developing predictive indexes. Furthermore, PFA systems differ substantially in electrode geometry, field distribution, and delivery modality, necessitating system-specific validation of any proposed PFA index. For example, the influence of contact force may vary according to catheter form factor (focal, multielectrode, or large-area electrodes) and delivery configuration (monopolar vs. bipolar).^37^ Most published PFA indexes to date have been derived from monopolar systems, and their applicability to bipolar systems remains uncertain. In bipolar configurations, factors such as catheter orientation may exert a greater influence on lesion formation due to differences in electric field confinement and current pathways (*Figure 5*).

**Figure 5:**
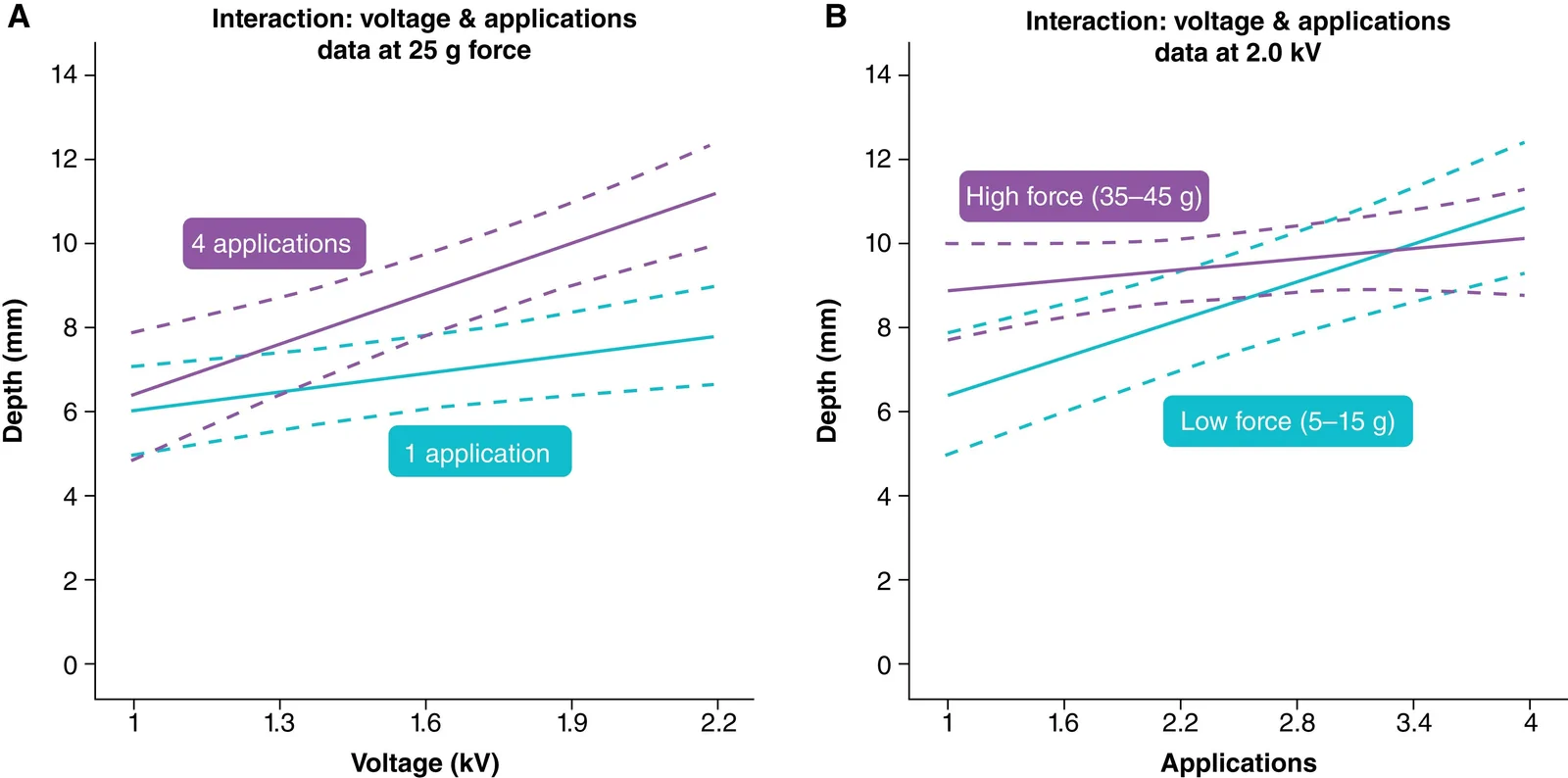
Two-way interactions between voltage, number of applications, and contact force and their effect on lesion depth. Among voltage, number of applications, and CF, two separate interactions were identified as having an effect on lesion depth. *A*) Examines the interaction between voltage and the number of applications at a fixed CF of 25 g, while *B*) evaluates the interaction between CF and the number of applications at a fixed voltage of 2.0 kV. *A*) At low voltages, the number of applications has little effect on lesion depth. However, at higher voltages, lesion depth increases with more applications. The lines indicate the predicted lesion depth for four applications and one application across varying voltage levels. *B*) With only one application, contact force strongly influences lesion depth, with higher force (35–45 g) producing deeper lesions than lower force (5–15 g). However, when four applications are applied, the effect of contact force on lesion depth is reduced. Adapted from Younis *et al*. Factors influencing lesion titration in a monopolar PFA point catheter: a preclinical study, Circ Arrhythm Electrophysiol. 2026;19:e014157. CF, contact force; PFA, pulsed field ablation

Multiparametric estimators are calculated values primarily derived from ablation inputs and do not directly measure tissue response. In RFA, local impedance serves as a surrogate of tissue coupling and characteristics and has been shown to independently predict lesion efficacy and durability.^73^

Ivorra *et al*. described the dynamics of tissue impedance changes following PFA in rat liver, suggesting that impedance variations may differentiate reversible from irreversible electroporation. However, the complex temporal evolution of impedance after PFA and the need for measurements at multiple time points limit its feasibility in routine clinical practice.^74^

Similarly, several studies have evaluated the temporal evolution of unipolar electrograms and demonstrated their potential to distinguish reversibly from irreversibly electroporated tissue.^75,76^ Nevertheless, this approach also requires repeated measurements at different time points and dedicated software algorithms to ensure consistent analysis of amplitude and slope changes.

Finally, while quantitative indexes may support lesion assessment and procedural standardization, they should be integrated with clear manufacturer-provided workflow recommendations, including interlesion distance guidance, to ensure lesion contiguity and procedural consistency. Standardized workflows remain critical to achieving durable lesion sets, irrespective of the ablation modality.

### Electrogram-based feedback

Decades of experience with RFA have allowed a better understanding of the acute behaviour of intracardiac electrograms. Local electrogram amplitude reduction is a well-recognized electrophysiological marker of effective thermal lesion formation, as well as impedance drop and pacing threshold increase. However, attenuation—or even complete abolition—of local electrograms and pacing threshold increase immediately after PFA does not necessarily correlate with durable myocardial lesion formation. Additionally, the details of impedance change before, during and after PFA are incompletely understood.

Tissue injury induced by electroporation is mediated by increased cell membrane permeability resulting from the formation of nanoscale pores within the lipid bilayer in response to the application of a high-intensity electric field. The size, density, and persistence of these pores are determined by the magnitude of the applied electric field, which may reach a critical threshold of irreversibility, beyond which durable cellular injury and cell death occur.^77^ When this threshold is not exceeded, cells ultimately recover, and the effects of electroporation may remain transient and fully reversible. Reversible electroporation should not be confused with exposure to electric fields below the electroporation threshold. Rather, it occurs when the applied electric field exceeds the threshold required to induce membrane permeabilization but remains below that required to produce irreversible cell injury. At lower field strengths, below the electroporation threshold, membrane permeabilization does not occur, and tissue remains electrically and structurally unaffected. The cellular repair mechanisms resulting in either membrane sealing or late cell death in humans are complex and incompletely understood.

At the cellular level, reversible electroporation is acutely associated with a pronounced but transient dysregulation of Ca^2+^ homeostasis, which gradually returns to baseline over minutes. Consistent with these acute cellular mechanisms, myocardial excitation–contraction coupling may be markedly impaired or temporarily abolished for several minutes.^78^ Early preclinical studies evaluating intracavitary electrogram behaviour after PFA have shown a significant acute reduction in local electrogram amplitude, followed by progressive recovery over time in regions exposed to reversible electroporation.^79^

The ability to distinguish acutely between transient impairment of cardiomyocyte function and irreversible electroporation-induced injury is therefore critical for accurately assessing lesion efficacy during ablation procedures. To this end, several experimental studies have analysed the acute temporal evolution and morphological characteristics of intracardiac electrograms following PFA in swine models, correlating these findings with lesion development over time.

#### Acute electrogram changes and their limitations

The main acute changes in electrogram morphology observed in both ventricular and atrial tissue following PFA are shown in *Figure 6*: (i) a significant reduction in bipolar and unipolar peak-to-peak amplitude (R/S-wave), (ii) marked ST-segment elevation on unipolar electrograms, and (iii) a pronounced decrease in |(dV/dt)|max.^31,75,80^

- Onset and magnitude of electrogram changes: All electrogram changes occur more rapidly after PFA than after RFA.^75,81^ The most pronounced morphological changes following PFA are observed within the first 2 min^31,75^ However, the magnitude of acute electrogram changes observed in both the atrium and the ventricle does not reliably predict lesion durability or transmurality.^75,82^
- Electrogram recovery dynamics: Following PFA, electrogram parameters exhibit dynamic short- and long-term recovery over time. In the ventricle, the ST segment progressively returns towards baseline within the first 30 min of observation, whereas unipolar peak-to-peak amplitude and |(dV/dt)|max gradually increase but remain significantly below baseline acutely. Following RFA, the initial recovery phase is also slower: although ST-segment elevation gradually decreases, electrogram amplitude and |(dV/dt)|max remain largely stable throughout the observation period.^75^

The magnitude and temporal evolution of electrogram changes after PFA are primarily determined by PFA parameters—particularly the applied voltage—and by the resulting electric field strength, which decreases with increasing distance from the catheter.^80^ Although PFA affects a relatively wide area of tissue surrounding the catheter, electrogram changes in the periphery—corresponding to regions of reversible injury or penumbra—are less sustained and recover more rapidly than those in the central lesion core. By comparison, electrogram alterations after RFA are largely confined to the central ablation zone, with minimal or negligible changes in the surrounding tissue.
In the atrium, PFA transmural lesions show minimal changes in bipolar signals during the first 10 min, whereas non-transmural lesions exhibit rapid recovery, reaching a plateau at ∼3.5 min. Similarly, ST-segment recovery on unipolar recordings occurs earlier and more steeply in non-transmural lesions. In contrast, lesions created by RFA do not display a significant recovery phase, regardless of transmurality.^82^

- Predictability of lesion size and durability: Several preclinical studies have correlated electrogram changes—both in magnitude and recovery dynamics—with histological findings to explore whether acute cut-off values could help to identify non-durable lesions after PFA. These thresholds have typically been established within the first 5 min in the atrium and 30 min in the ventricle.^75,82^ However, although differences between irreversibly and reversibly electroporated tissue are evident, accurate delineation of lesion borders remains challenging because electrogram behaviour in the transitional zone is intermediate.^75^

**Figure 6:**
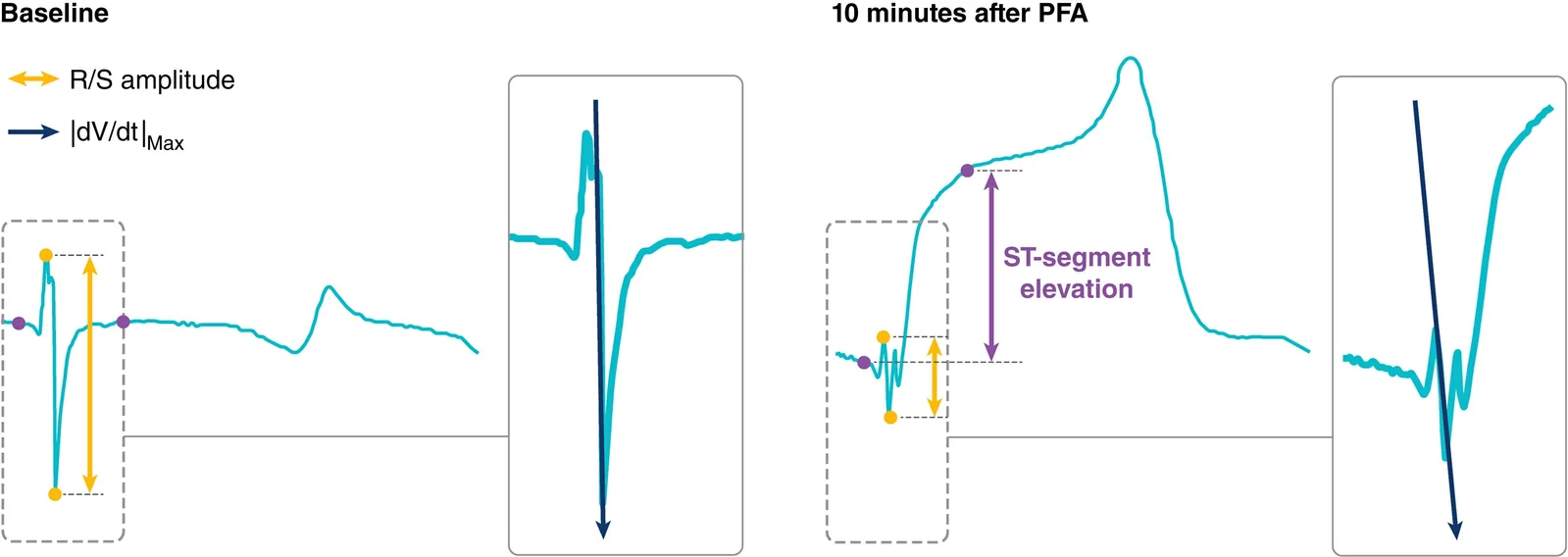
Main acute electrogram morphology changes observed immediately after PFA delivery. Adapted from Amorós-Figueras G, *et al*. Dynamics of High-Density Unipolar Epicardial Electrograms During PFA. Circ Arrhythm Electrophysiol. 2023 Sep;16(9):e011914. PFA, pulsed field ablation

#### Subacute lesion durability and limits of acute electrogram assessment

Beyond the acute effects of electroporation, the subacute evolution of ablation injury remains poorly characterized and incompletely understood. The cellular repair mechanisms and their temporal course that may lead to partial lesion recovery, pulmonary vein reconnection, and ablation failure occur with both RFA and PFA, yet are only weakly reflected by acutely observed electrogram changes.

In humans, clinical evidence suggests that delayed lesion recovery occurs with all currently available PF devices, as dose-finding studies required waveform optimization to reduce high pulmonary vein reconnection rates at obligate remapping, and even with optimized parameters, the MANIFEST redo study demonstrated durable pulmonary vein isolation in only 45% of patients. Consistent with these clinical observations, the correlation between acute electrogram changes and subacute lesion durability or morphology is limited. In ventricular tissue, only modest associations have been reported between unipolar amplitude reduction or ST-segment elevation and lesion depth or volume, while in atrial tissue, correlations with lesion transmurality are similarly weak and confined to specific frequency components of bipolar and unipolar electrograms in preclinical models.^31,80,82^

Despite being difficult to investigate even in animal studies, further research is needed to better characterize subacute cellular repair and lesion recovery, and to improve the ability of acute electrogram changes to predict subacute lesion durability.

#### Reversible pulsed-field mapping: translating transient electrogram changes into definitive lesion strategy

Transient changes in electrograms and cardiomyocyte conduction induced by reversible PFA may be advantageous for delineating complex arrhythmia circuits and identifying the optimal ablation target before creating definitive lesions. In this context, both preclinical and clinical experience have demonstrated the feasibility and high reproducibility of non-triggered pulsed-field (PF) mapping as a novel mapping tool for the evaluation of atrial flutter re-entrant circuits.^79,83^ It should be noted, however, that the cellular behaviour in the reversible penumbra around irreversible electroporation may significantly differ from very short-duration reversible electroporation used for mapping.

Application of a single reversible PF pulse to a given circuit can elicit four distinct electrophysiological responses: (i) tachycardia termination, (ii) stable prolongation of the tachycardia cycle length (TCL) with preservation of the activation pattern, (iii) irregular TCL variations, and (iv) no observable change. Notably, only the first two responses are observed when the pulse is delivered at a critical site, within the circuit, with a reported specificity of 100%. These effects are attributed to reversible electroporation transiently disrupting cardiomyocyte excitation–contraction coupling, either by suppressing conduction and terminating the tachycardia or by slowing conduction, resulting in TCL prolongation.^83^ Representative examples are shown in *Figure 7*.

**Figure 7:**
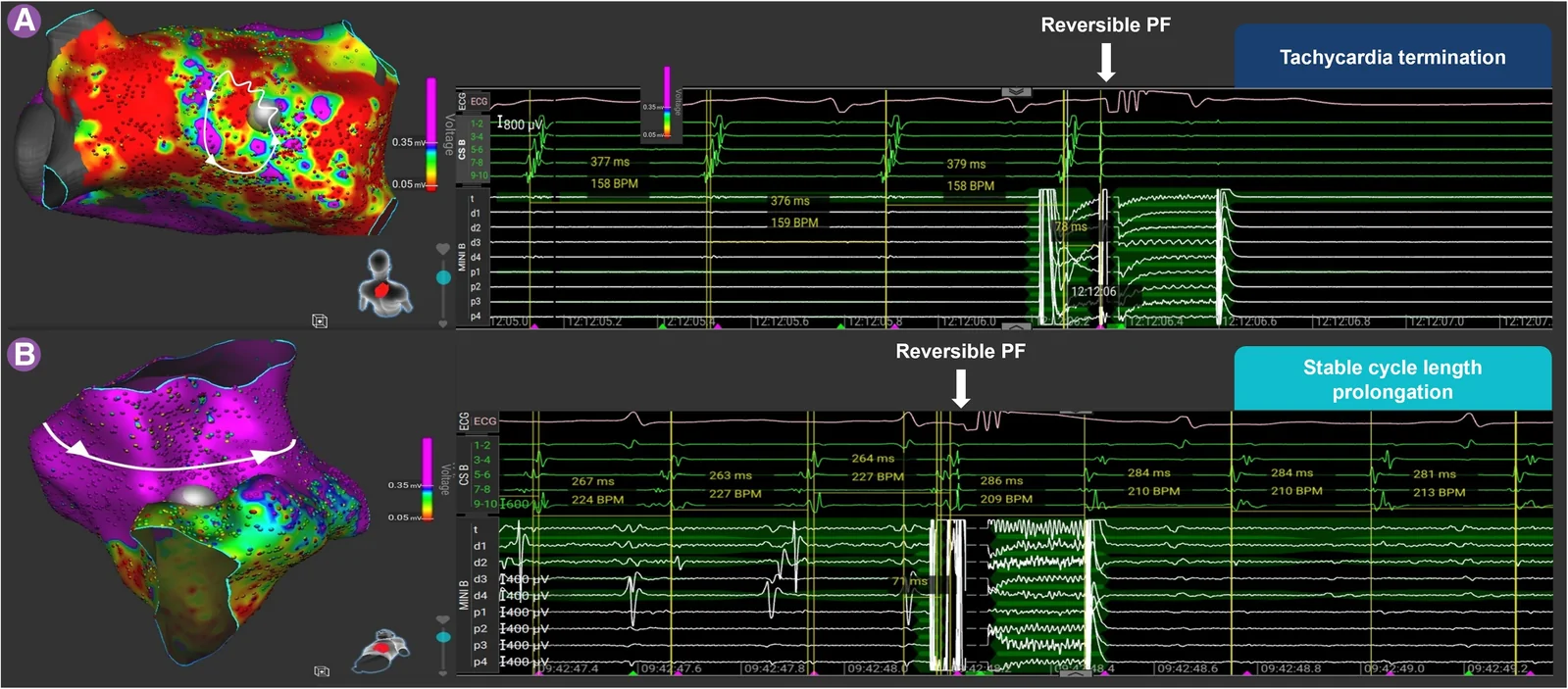
Illustrative examples of specific electrophysiological responses to reversible PF delivered at the critical isthmus. *A*) Non-propagated reversible PF delivered at the left atrial posterior wall leading to tachycardia termination. *B*) Non-propagated reversible PF delivered at cavotricuspid isthmus leading to stable tachycardia cycle length prolongation. PF, pulsed-field

At present, however, this strategy demonstrates only moderate sensitivity. Accordingly, reversible PF mapping should currently be viewed as an investigational mapping concept rather than an established procedural strategy, pending validation in larger clinical studies. This limitation is largely related to the lack of dose-titration capabilities in current clinical systems and to the influence of critical isthmus properties on the electrophysiological response to reversible PF delivery.

## Emerging concepts and future directions

Emerging technologies in PFA increasingly aim not only to improve lesion depth but also to provide reliable intraprocedural assessment of treatment efficacy, addressing one of the major limitations of non-thermal ablation.^84,85^

Some catheters are combining both PFA and thermal energy to try to increase performance. The short-duration ultra-low-temperature catheter from Adagio Medical coats the catheter with ice and pre-freezes targeted myocardial tissue, focusing pulsed electric field to the frozen areas and reducing aberrant electric currents elsewhere. Clinically, this approach is intended to help minimize skeletal muscle contraction, phrenic nerve capture, and microbubbles (Adagio Medical). Similarly, sequential radiofrequency and PFA delivery has been investigated to create deeper lesions,^86^ although results remain inconsistent and sometimes contradictory,^87,88^ with several observations suggesting no clear advantage compared with optimized PFA alone. In contrast, repeated PFA applications (‘PF + PF’) appear capable of enlarging lesions through cumulative membrane destabilization and prolonged pore persistence, achieving deeper effects without introducing additional thermal injury.

Parallel developments focus on acute lesion assessment by implementing automated or AI-guided tools to analyse the electrical activity of the tissue after PFA. Tools such as the PFAanalyzer tool (CathVision) can interpret remaining unipolar electrograms and detect residual conduction through advanced signal characterization. Some other developments are focused on tissue mechanical and biophysical properties rather than electrical using optical mapping techniques. Polarization-sensitive imaging based on myocardial birefringence enables detection of microstructural alterations in cardiac fibres induced by electroporation (AblaView®, MedLumics), reflecting cytoskeletal disruption rather than conduction changes alone. Although these approaches are promising, there is currently insufficient clinical evidence to demonstrate that AI-assisted lesion assessment improves procedural outcomes or long-term durability.

Pulsed field ablation is likely to evolve towards a broader ecosystem of technologies, with new form factors, from focal to large-focal platforms, designed to address diverse indications across different cardiac chambers. The next major step for the field will probably be the adaptation of current technologies to ventricular applications, where achieving deep, durable lesions must be balanced with preservation of the conduction system, myocardial function, and coronary arteries. In parallel, the market will likely move towards the development of smarter procedural tools, such as lesion indexes, smart tags, or other feedback solutions, to enable real-time assessment of lesion performance and durability during the acute procedure (*Table 1*).

**Table 1:** Current and emerging approaches for intraprocedural assessment of lesion durability in pulsed field ablation

Technology/marker	Signal/principle	Intended purpose	Development stage/evidence	Implementation/limitations
Electrogram recovery dynamics	Time-dependent evolution of bipolar/unipolar electrograms after PFA	Differentiate reversible from irreversible electroporation	Preclinical + emerging clinical evidence^75,76,82^	Requires prolonged intracardiac recordings at fixed sites (minutes-scale monitoring); limited feasibility for real-time workflow integration; complex signal interpretation
Impedance-based metrics	Electrical impedance variation during and after PFA delivery	Infer tissue injury and changes in conductivity	Preclinical^74,89^	Highly system-dependent; lack of standardized thresholds; limited specificity for lesion durability
Contact force	Mechanical coupling of catheter tip with tissue	Improve consistency of energy delivery and lesion reproducibility	Preclinical/clinical^37,44,71,90^	Depends highly on catheter form factor. Correlation with lesion depth only demonstrated for small tip focal-monopolar catheters.
Composite deterministic ablation indices	Multivariable models incorporating CF, voltage, pulse number, and related parameters	Estimate lesion geometry (depth and volume)	Preclinical^30^	Limited generalizability across PFA systems; no validated clinical endpoint correlation
AI-based electrogram analysis	Morphology and temporal evolution of unipolar intracardiac electrograms	Detect residual conduction or incomplete lesion formation	Early development	Lack of validated association with chronic lesion durability; requires standardized datasets and prospective validation
Intracardiac echocardiography (ICE)—tissue characterization	Echogenicity changes (‘tissue brightening’), interface visualization	Indirect assessment of acute tissue reaction after PFA	Clinical observational	No standardized criteria for lesion definition; interpretation is operator- and system-dependent; not a direct lesion quantification tool
Optical mapping	Optical/birefringence-based detection of myocardial microstructural changes	Detection of electroporation-induced structural alterations	Preclinical/early translational^91^	Limited clinical availability; requires dedicated hardware; not validated for prediction of chronic lesion durability. Requires prolonged procedure time to analyze lesions
Catheter shadow projection in 3D electroanatomical map	Spatial tagging of catheter position and energy delivery	Ensure lesion coverage and continuity	Clinical	Purely anatomical/positional tracking; no direct information on tissue injury
Estimated electric field intersection in 3D electroanatomical map	Field-integrated tagging based on catheter position and estimated electric field exposure	Estimate lesion extent and predict chronic lesion size	Preclinical + clinical validation studies^7^	Promising correlation with chronic lesion size; system-specific implementation, anatomy-dependent; requires external validation across platforms

Summary of current technologies and procedural markers used or under investigation to assess lesion formation and predict chronic lesion durability during PFA. Technologies are categorized according to their underlying signal or measurement principle, intended clinical application, current level of evidence, and major implementation limitations. Importantly, no currently available approach has been universally validated to reliably predict chronic lesion durability during the acute procedure across different PFA platforms.

Abbreviations: AI, artificial intelligence; CF, contact force; ICE, intracardiac echocardiography; PFA, pulsed field ablation.

Evidence level: *Preclinical* indicates evidence derived primarily from experimental or animal studies. *Clinical observational* indicates evaluation in human studies without prospective validation of chronic lesion durability. *Clinical* refers to technologies currently used in routine clinical practice, although not necessarily validated as predictors of durable lesion formation. *Early development* denotes investigational technologies that remain under active development or early clinical evaluation.

## Data Availability

The data underlying this article will be shared on reasonable request to the corresponding author.
